# Efficiency of genomic prediction for boar taint reduction in Danish Landrace pigs

**DOI:** 10.1111/age.12369

**Published:** 2015-10-09

**Authors:** B. Lukić, R. Pong‐Wong, S. J. Rowe, D. J. de Koning, I. Velander, C. S. Haley, A. L. Archibald, J. A. Woolliams

**Affiliations:** ^1^ Faculty of Agriculture in OsijekJ.J. Strossmayer University of OsijekKralja Petra Svačića 1d31000OsijekCroatia; ^2^ The Roslin Institute and R(D)SVSUniversity of EdinburghEaster BushMidlothianEH25 9RGUK; ^3^ Swedish University of Agricultural SciencesSE‐750 07UppsalaSweden; ^4^ Pig Research CentreDanish Agriculture & Food CouncilAxeltorv 3KøbenhavnV 1609Denmark; ^5^ MRC Human Genetics UnitMRC IGMMUniversity of EdinburghCrewe RoadEdinburghEH4 2XUUK

**Keywords:** androstenone, Bayes, genomic best linear unbiased prediction, genomic selection, skatole

## Abstract

Genetic selection against boar taint, which is caused by high skatole and androstenone concentrations in fat, is a more acceptable alternative than is the current practice of castration. Genomic predictors offer an opportunity to overcome the limitations of such selection caused by the phenotype being expressed only in males at slaughter, and this study evaluated different approaches to obtain such predictors. Samples from 1000 pigs were included in a design which was dominated by 421 sib pairs, each pair having one animal with high and one with low skatole concentration (≥0.3 μg/g). All samples were measured for both skatole and androstenone and genotyped using the Illumina SNP60 porcine BeadChip for 62 153 single nucleotide polymorphisms. The accuracy of predicting phenotypes was assessed by cross‐validation using six different genomic evaluation methods: genomic best linear unbiased prediction (GBLUP) and five Bayesian regression methods. In addition, this was compared to the accuracy of predictions using only QTL that showed genome‐wide significance. The range of accuracies obtained by different prediction methods was narrow for androstenone, between 0.29 (Bayes Lasso) and 0.31 (Bayes B), and wider for skatole, between 0.21 (GBLUP) and 0.26 (Bayes SSVS). Relative accuracies, corrected for *h* ^2^, were 0.54–0.56 and 0.75–0.94 for androstenone and skatole respectively. The whole‐genome evaluation methods gave greater accuracy than using only the QTL detected in the data. The results demonstrate that GBLUP for androstenone is the simplest genomic technology to implement and was also close to the most accurate method. More specialised models may be preferable for skatole.

## Introduction

Androstenone and skatole are compounds that accumulate in the fat of mature non‐castrated male pigs. This accumulation results in an offensive odour, called boar taint, that affects the smell and taste of cooked pork (Babol *et al*. 1999). Androstenone is a testicular steroid which produces a urine‐like odour, whereas skatole, a product from the breakdown of tryptophan by microbial activity in the intestine, exhibits a faecal odour in pork. In the EU, castration is commonly used to avoid boar taint with different approaches varying across countries (Fredriksen *et al*. 2009). However, a voluntary agreement initiated by the European Commission (2011) declared that castration in pig production should be eliminated by 2018 due to social pressure and animal welfare issues. Several alternative approaches have been proposed for preventing boar taint (Bonneau & Squires 2004). For example, immunocastration is one alternative, involving vaccination to inhibit testicular function, but problems arise due to cost (de Roest *et al*. 2009), the need for repeated vaccinations (Squires & Bonneau 2004) and variation in vaccine response (Bonneau *et al*. 1994; Turkstra *et al*. 2002), and there are risks to male operatives from accidental self‐inoculation. Other alternatives include slaughtering animals before sexual maturity, which is a common practice in the UK but is not acceptable in most EU countries for reasons of consumer acceptability or profitability (Xue & Dial 1997). A more acceptable and practical long‐term approach is the genetic selection of animals against expression of boar taint (Quintanilla *et al*. 2003; Lee *et al*. 2005; Moe *et al*. 2009; Duijvesteijn *et al*. 2010; Squires & Schenkel 2010; Rowe *et al*. 2014).

Evidence for genetic variation in androstenone and skatole concentrations in fat tissue has been reported in numerous studies amongst breeds (Duijvesteijn *et al*. 2010; Le Mignon *et al*. 2010; Grindflek *et al*. 2011; Robic *et al*. 2011; Gregersen *et al*. 2012). Within‐breed estimates of heritability range from 0.25 to 0.88 for androstenone and 0.19 to 0.54 for skatole (reviewed by Robic *et al*. 2008). However, exploiting this variation is challenging, as the trait is age‐limited, sex‐limited and destructive: only males express taint, it is not expressed until after sexual maturity and can be measured only after slaughter (excluding invasive techniques). One approach to overcome all these challenges is the use of genomic predictors, available from birth in both sexes and, with adequate training data, capable of delivering high accuracy. Such predictors may be based either upon a handful of causative mutations explaining a high proportion of the variance or via genomic evaluation (Meuwissen *et al*. 2001). To date, genomic selection has been widely applied to livestock production in cattle, pigs and poultry.

There has been little consensus in the literature regarding the genetic architecture of boar taint. QTL mapping studies and genome‐wide association studies (GWASs) appear to have identified QTL that differ markedly by location and effect (Quintanilla *et al*. 2003; Lee *et al*. 2005; Grindflek *et al*. 2011; Rowe *et al*. 2014). The reason for this may be the different breeds that were used, or this could indicate that many genes have an effect. The genetic architecture influences the effectiveness and accuracy of different methods of genomic evaluation (Daetwyler *et al*. 2010). Two of the most commonly used methodologies for genomic evaluation are genomic best linear unbiased prediction (GBLUP) and Bayesian approaches which assume various priors in which some subsets of markers are assumed to explain more variance than others. The latter is advantageous when the number of QTL explaining the variance is small (Daetwyler *et al*. 2010), and a number of Bayesian regression methods have been proposed that differ in their assumptions for partitioning SNPs into those with ‘large’ vs. ‘small’ effects and the distributional assumptions within these classes.

The aim of this study was to assess the potential for genomic selection on two compounds related to boar taint – skatole and androstenone – by assessing the prediction accuracy of GBLUP and five regression‐based Bayesian methodologies. The study was made feasible by the availability of a large case–control data set on commercial pigs obtained from abattoirs. The data included information on both skatole and androstenone for which, as indicated above, preliminary evidence has suggested differences in genetic architecture.

## Materials and methods

### Animals

All the animals involved in this study were raised under conventional pig production conditions and were not subjected to any experimental procedures. All samples for the study were collected post‐mortem in a commercial abattoir.

### Sample collection

Samples were collected at the abattoir from 6178 intact male Danish Landrace pigs of known pedigree and known farm of origin. Two samples of adipose tissue were collected from each animal at the abattoir: the first immediately after the carcass was cut into two sides and the second one hour later. The first samples were assayed immediately for skatole levels in‐house at the abattoir, as these were used to select animals for genotyping and androstenone measurement, as described below. The second adipose sample for determining androstenone and a muscle sample for DNA extraction from each animal were stored at −20 °C.

### Selection of animals for analysis

Skatole concentrations (μg/g fat tissue) were measured using a spectrophotometric method (Møller & Andersen 1994) and were used to select 1000 animals in a sib‐pair design. Five hundred animals with high skatole concentrations in fat tissue (≥0.3 μg/g) were selected. For each selected individual, the available littermate with the lowest skatole concentration, always with a skatole concentration of <0.3 μg/g fat tissue, was selected as a control (in a few cases, when due to unexpected experimental errors it was not possible to sample a littermate to genotype, a corresponding control animal was selected from another litter). The concentration of androstenone in fat tissue (μg/g) was then measured in the 1000 selected animals by time‐resolved fluoro‐immunoassay, as described by Tuomola *et al*. (1997), modified using antiserum produced and characterised by Andresen (1974). Chemical analyses of skatole and androstenone were performed at Landbrug & Fødevarer (Denmark) and the Norwegian School of Veterinary Science respectively.

The 1000 selected animals were then genotyped for 62 153 SNPs using the Illumina PorcineSNP60 BeadChip (Ramos *et al*. 2009). SNP loci with minor allele frequencies (MAF) ≤ 0.01, call rate ≤ 0.95 and extreme departure from Hardy–Weinberg equilibrium when assessed using a false discovery rate of 1% were removed. These criteria removed 13 795, 3217 and 678 SNPs respectively. Individuals with a call rate ≤ 0.95 and autosomal heterozygosity ≥ 0.45 were then removed. An extremely high relationship between two individuals may indicate that they are twins but also may indicate an error due to duplicate samples, so any pair of animals showing a relationship ≥ 0.95 was eliminated from the analysis. After quality control, 938 males with data on 42 916 SNPs (69%) remained. The 938 intact males comprised: 842 animals from sib pairs (421 pairs), with each pair having one animal with a high and one with a low skatole concentration; 40 animals with high skatole concentrations with no littermate; and 56 animals with low skatole concentrations with no littermate. The animals with no full‐sib littermates had paternal half‐sibs in other litters. In total, the 461 cases and 477 controls had been bred from 128 sires and 441 dams and had been reared on 14 farms.

In addition, the littermate design confirmed the expected population stratification due to the presence of closely related individuals. A clustering model was computed with the mclust function in r software 2.10, and multidimensional scaling (MDS) was performed resulting in individuals being grouped into three clusters (Rowe *et al*. 2014), which separated some sire families. However, there was no structural confounding observed between these clusters and the high and low skatole concentration groups because of the procedure for sampling animals for genotypes. This was confirmed in preliminary analyses by fitting the clusters as an independent factor in a linear model and observing no significant effect.

### Available data

Information that was collected on each of the 938 animals included: sire, dam, age at slaughter, cold carcass weight, meat percentage and the farm of rearing. The average age of selected animals at slaughter was 161.3 days (SD = 1.36), and the average cold carcass weight was 77.34 kg (SD = 9.47). Average meat percentage was 60.13% (SD = 3.18), determined by the standard Danish classification system in slaughterhouses.

### Methods of analyses

Phenotypic values for both traits were pre‐corrected for farm as a fixed effect and age as a covariate prior to genetic analysis (they were shown to be significantly affecting both traits by Rowe *et al*. 2014). Meat percentage and cold carcass weight were not used as covariates, as they could be confounded with genes that affect boar taint. The log‐transformation was applied for skatole and androstenone phenotypic values to more closely approximate normal distributions. Six different models, GBLUP and five Bayesian variants, were fitted to both androstenone and skatole, as described below.

#### GBLUP

A mixed linear model was fitted as follows:

y=μ1+u+e,

where **y** is a vector of phenotypes of the trait; *μ* is the mean; **1** is vector of ones; **u** is a vector of random additive genetic effects assumed to be distributed MVN (0, $\sigma_{g}^{2}G$), where **G** is a relationship matrix computed from the SNP information and constructed following Amin *et al*. (2007) and $\sigma_{g}^{2}$ is the associated variance; and **e** is the vector of residuals assumed to be distributed MVN (0, $\sigma_{e}^{2}I$), where **I** is the identity matrix. Amin *et al*. (2007) calculate **G** by:

$$g_{\text{ij}}=n^{-1}\sum_{k=1}^{n}\left(x_{\text{ik}}-2p_{k}\right)\left(x_{\text{jk}}-2p_{k}\right)/\left[2p_{k}\left(1-p_{k}\right)\right]$$

and

$$g_{\text{ii}}=1+n^{-1}\sum_{k=1}^{n}\left(H_{E,k}-H_{\text{ik}}\right)/H_{E,k},$$

where *g*_ij_ is the genomic relationship between animals *i* and *j*;* x*_IK_ is the genotype of the *i*th individual at the *k*th SNP when coded as 0, 1 and 2 for the reference allele homozygote, the heterozygote and alternative homozygote respectively; *p*_*k*_ is the frequency of the reference allele; *n* is the number of SNPs used for estimating relationships; *H*_*E,k*_ is the expected heterozygosity at locus *k*; and *H*_ik_ is the observed heterozygosity in animal *i* at locus *k*. This model was fitted using asreml 3.0 (Gilmour *et al*. 2000).

#### Bayesian regression methods

The linear model fitted for these methods was the following:

y=μ1+Zβ+e,

where **y** is the vector of phenotypes; *μ* is overall mean for the trait; **1** is vector of ones; **Z** is the matrix of genotypes, where *z*_ik_ is the number of alternative alleles for individual *i* at SNP locus *k*;***β*** is a vector of regression coefficients, where *β*_*k*_ is the coefficient for SNP locus *k*; and **e** is the vector of residuals assumed to be distributed MVN (0, $\sigma_{e}^{2}I$). The *β*_*k*_ values are assumed to be independent random variables drawn from prior distributions which differ amongst the five Bayesian models.

The five models and their associated priors are as follows:

1. Bayes A: The prior distribution for *β*_*k*_ is a scaled Student's *t* distribution with two parameters scale, *λ* and shape *υ*.
2. Bayes B: As Bayes A but where only a fraction *π* of SNPs have effects from the scaled Student's *t* distribution (with parameters scale *λ* and shape *υ*) with the remaining (1–*π*) having a zero effect.
3. Bayes C: Similar to Bayes B but with non‐zero effects assumed to be normally distributed with variance $\sigma_{s}^{2}$ instead of the scaled Student's *t* distribution and with the mixing parameter *π*.
4. Bayes SSVS: Similar to Bayes C but with effects coming from a mixture distribution of two normal distributions, one with variance $\sigma_{s}^{2}$ and the other with variance $\sigma_{s}^{2}$/10 000 and mixing parameter *π* (see Verbyla *et al*. 2009).
5. Bayesian Lasso: Similar to Bayes A, but a Laplace distribution with scale parameter *λ* replaces the scaled Student's *t* distribution.

Frequently, the different parameters defining the prior distributions of *β*_*k*_ have been assumed as hyperparameters and fixed in the analysis to a value preset by the researcher (e.g. Meuwissen *et al*. 2001; Hayes *et al*. 2009). Here, these parameters were included in the analysis and estimated from the data, with the exception that *π* as the low heritability of skatole made the analysis prone to convergence problems when using Bayes C, where it was fixed to be 0.1, but preliminary analysis showed that the results were similar over a range of small values for *π*. For all the other parameters defining the distributions of SNP effects, a bounded flat prior was assumed. The scale parameter *λ* (included in Bayes A, Bayes B and Bayesian Lasso), the variance parameter $\sigma_{s}^{2}$ (included in Bayes C and Bayes SSVS) and the residual variance $\sigma_{e}^{2}$ were all bounded between 0 and a very large positive number so that any influence of the prior on the estimated genetic variance was negligible. The shape parameter *υ* in Bayes A and Bayes B were bounded between 0.5 and 8.

The implementation of the Bayesian regression method was carried out using Gibbs sampling. For each of the analysis carried out here, the first 50 000 cycles of the Monte Carlo Markov chain were discarded as a burn‐in period. Results were calculated from a minimum of 20 000 subsequent realisations where consecutive realisation was separated by 50 cycles. The whole chain therefore consisted of 1 050 000 cycles.

### Calculation of heritabilities

Heritability was estimated as $h^{2}=\sigma_{g}^{2}/\left(\sigma_{g}^{2}+\sigma_{e}^{2}\right)$. For GBLUP, the estimate of $\sigma_{g}^{2}$ was estimated directly in the analysis. For Bayesian regression methods, $\sigma_{g}^{2}$ was calculated following Nadaf *et al*. (2012), in which the estimate of $\sigma_{g}^{2}$ was obtained from $\sigma_{g}^{2}=var\left(EBV\right)+\bar{PEV}$, where $\bar{PEV}$ is the average prediction error variance in the training population. $\bar{PEV}$ was calculated from the Gibbs chain. In the results, $\sigma_{e}^{2}$ for each model is also presented, which represents that part of the phenotypic variance that remains unexplained by the genetic model.

### Cross‐validation and comparisons between the methods

A fivefold cross‐validation was carried out to compare the accuracy of GBLUP and the five Bayesian regression methods – Bayes A, Bayes B, Bayes C, Bayes SSVS and Bayesian Lasso – to predict the unobserved phenotypes. The division of the full data set preserved sib pairs but was otherwise randomly separated into five cross‐validation sets resulting in training sets of ~751 animals and validation sets of ~187 animals. Each training set had a size of approximately 4/5 of the whole data set with phenotypes and each animal appearing in precisely one validation set. For each training set, GBLUP and Bayesian regression methods were used to estimate genomic estimated breeding values (GEBVs) and heritabilities. Accuracy (*r*) in predicting the phenotype was calculated as the correlation between the GEBV and the phenotypes of validation animals, and the overall values of accuracies were calculated as the average over the five validation sets. Principal component analyses (PCAs) for both traits were performed to show the relative relationship between all the methods investigated.

### Comparisons with QTL

The difference between genomic predictions using all SNPs and an approach utilising only SNPs identified from GWAS was assessed by calculating the predictive accuracy of all SNPs identified as statistically significant (*P *<* *0.05) genome‐wide from the same data set (Rowe *et al*. 2014). These SNPs were *H3GA00016037* on SSC5 for androstenone concentration and *SIRI0000194* on SSC14 for skatole concentration. This was done using the five cross‐validations sets with the phenotype of each set being predicted using estimates of the magnitude of the QTL effect derived by estimating allelic substitution effects by fitting SNP genotypes (coded as 0, 1 and 2) to the remaining data.

## Results

### Androstenone

The accuracies (average correlation between the GEBV and phenotypes across the validation sets) obtained by the different methodologies are shown in Table 1. The range of accuracies for predicting phenotype was narrow for androstenone, ranging only between 0.291 (Bayes Lasso) and 0.310 (Bayes B), 6% of the mean accuracy, and with no clear difference between GBLUP and Bayesian regression methodologies. The estimated *h* ^2^ also were narrow, ranging from 0.276 (Bayesian Lasso) to 0.307 (GBLUP). GBLUP also had the lowest $\sigma_{e}^{2}$, which is the most objective component for comparison as its magnitude does not depend on scaling assumptions, but the range of estimates was only 4% of their mean. Scaling all the accuracies of predicting phenotypes by the square root of the average *h* ^2^ indicated that the accuracy of predicting the breeding value was ~0.56.

**Table 1:** Genetic ($\sigma_{g}^{2}$) and residual ($\sigma_{e}^{2}$) variance components, heritabilities (*h* ^2^) and accuracies (*r* and r*) for androstenone concentration (μg/g fat tissue) estimated by different methodologies

Method	$\sigma_{g}^{2}$	$\sigma_{e}^{2}$	*h* ^2^	*r*	r*
GBLUP	0.149	0.333	0.307	0.298	0.555
Bayes A	0.141	0.343	0.287	0.301	0.559
Bayes B	0.137	0.347	0.276	0.310	0.577
Bayes SSVS	0.143	0.343	0.281	0.299	0.555
Bayes C	0.149	0.337	0.299	0.300	0.559
Bayesian LASSO	0.137	0.346	0.284	0.291	0.541

*r,* the accuracy of predicting the phenotype calculated as the correlation between the estimated breeding value and phenotype; r*, the accuracy of predicted the breeding value, obtained by scaling *r* by the square root of the average *h* ^2^ over all methods. The average standard error for values of *r* obtained from the cross‐validation was 0.031.

### Skatole

The heritabilities and accuracies calculated as correlations between the GEBVs and phenotypes of the validation animals from different methodologies are shown in Table 2. Compared to androstenone, the range of accuracies for predicting skatole fat concentrations was wider, between 0.214 (GBLUP) and 0.266 (Bayes SSVS and C), corresponding to 21% of the mean over all methods, with GBLUP appearing to be a low outlier. In contrast, the range in estimates of $\sigma_{e}^{2}$ was very similar to androstenone, corresponding to 4% of the mean estimate over methods. The estimated heritability was highest with the Bayes C method (0.106) and lowest with GBLUP (0.051). Using the average of the estimates, the accuracy of predicting the breeding value was 0.88.

**Table 2:** Genetic ($\sigma_{g}^{2}$) and residual ($\sigma_{e}^{2}$) variance components, heritabilities (*h* ^2^) and accuracies (*r* and r*) for skatole concentration (μg/g fat tissue) estimated by different methodologies

Method	$\sigma_{g}^{2}$	$\sigma_{e}^{2}$	*h* ^2^	*r*	r*
GBLUP	0.014	0.466	0.051	0.214	0.755
Bayes A	0.037	0.446	0.094	0.265	0.934
Bayes B	0.030	0.452	0.074	0.252	0.888
Bayes SSVS	0.039	0.446	0.087	0.266	0.940
Bayes C	0.037	0.447	0.106	0.266	0.938
Bayesian LASSO	0.028	0.457	0.068	0.230	0.812

*r,* the accuracy of predicting the phenotype calculated as the correlation between the estimated breeding value and phenotype; r*, the accuracy of predicted the breeding value, obtain by scaling *r* by the square root of the average *h* ^2^ over all methods. The average standard error for values of *r* obtained from the cross‐validation was 0.014.

### Comparison of methods

**Figure 1:**
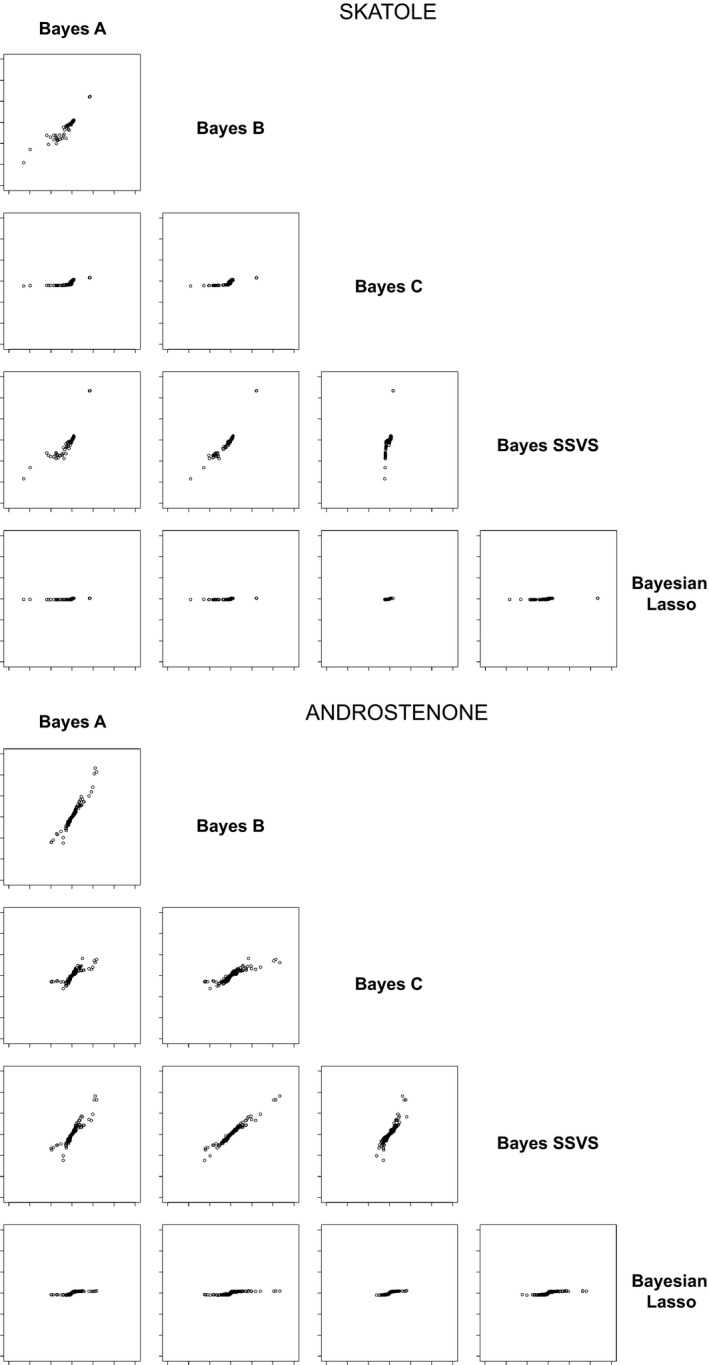
A comparison of estimated SNP effects, defined as the average value over realisations, obtained for five Bayesian regression methods. The upper plots correspond to skatole and the lower plots correspond to androstenone, both measured as μg/g fat tissue. Coordinate length for both *x* and *y* axes ranges from −0.03 to 0.03.

The relationships between individual SNP effects across methods are shown in Fig. 1. The plot confirms the strong similarity between Bayes B and Bayes SSVS and, in turn, their similarity with Bayes A. All three methods have the assumption that large SNP effects follow an inverse chi‐squared distribution. Bayes C shows a narrower range of values compared to these, as might be expected from the regularisation properties of these distributions. The SNP effects for Bayesian Lasso had the lowest variance of all methods.

For skatole, for which a single, strong QTL is present (Rowe *et al*. 2014), the best accuracy was obtained by Bayes SSVS followed by Bayes C. Bayesian Lasso performed similarly for both traits, achieving the lowest accuracy as well as capturing the lowest proportion of genetic variance.

**Figure 2:**
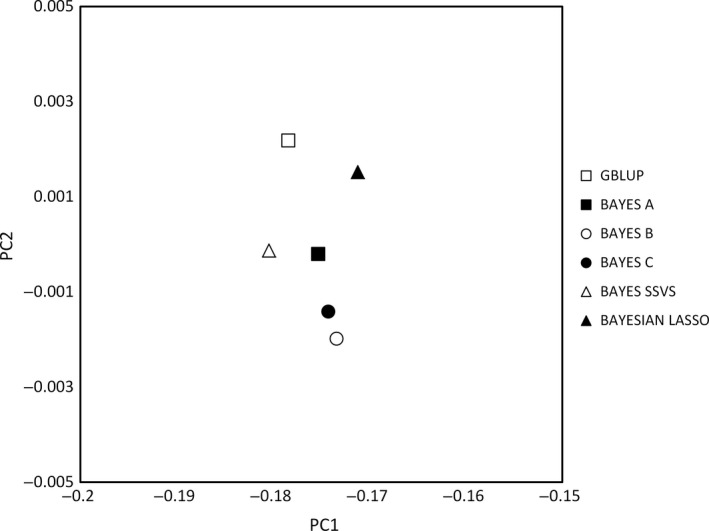
Scatterplot of the first two principal components (PC1 vs. PC2) on the GEBV for androstenone concentrations between all the methods. Each point represents a different method as follows: □ GBLUP, ■ Bayes A, ○ Bayes B, ● Bayes C, Δ Bayes SSVS, ▲ Bayesian Lasso.

**Figure 3:**
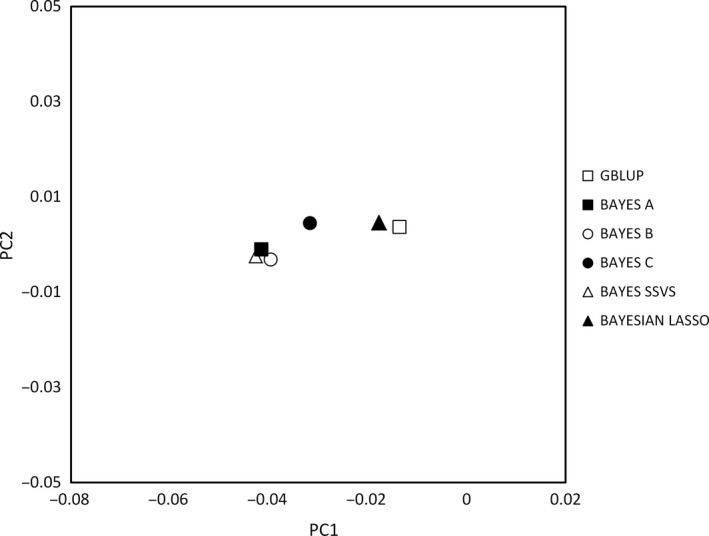
Scatterplot of the first two principal components (PC1 vs. PC2) on the genomic estimated breeding values for skatole concentrations amongst all the methods. Each point represents a different method as follows: □ GBLUP, ■ Bayes A, ○ Bayes B, ● Bayes C, Δ Bayes SSVS, ▲ Bayesian Lasso.

To further demonstrate relative relationships between the methodologies used, PCA was performed on GEBV, and the obtained results are presented in Figs 2 and 3. As expected, the scatter plot indicates greater similarity amongst the methodologies for the estimation of GEBVs for androstenone than for skatole. This relative similarity of the different methods was confirmed with the PCA of the GEBVs (see Figs 2 and 3). For both androstenone and skatole, Bayes A, B and SSVS tended to cluster together and Bayesian Lasso clustered with GBLUP, but the differences were small.

### Comparison with QTL

For androstenone, the accuracy of predicting phenotypes from the single significant SNP was 0.15, notably lower than the other genomic predictions using all SNPs. For skatole concentration, the accuracy in predicting phenotypes from the single genome‐wide significant SNP was 0.21, similar to GBLUP but lower than those obtained using Bayesian regression methodologies.

## Discussion

To our knowledge, this is the first study to test the different methodologies for genomic evaluation of androstenone and skatole concentrations – two compounds that are the directly related to the occurrence of boar taint – in the fat of slaughtered male pigs. It was shown that training data using all markers simultaneously in genomic evaluations (Meuwissen *et al*. 2001) produced better accuracies than using detected QTL. In the case of androstenone concentration, the accuracies obtained from GBLUP and a series of Bayesian regression methods were very similar. In contrast, for skatole concentration, where it has been established that a large QTL, explaining 77% of the genetic variance (Rowe *et al*. 2014) is segregating within this population, Bayesian regression methods fitting models where only a subset of SNP have large effects gave more accurate predictions than did GBLUP. However, such a benefit would not be expected for breeds in which this QTL is not segregating. In the situation for which a QTL with large effect has been mapped, the performance of GBLUP may be increased if the QTL is included in the model as a fixed effect, but if the mapped QTL turns out to be a false positive, the consequences for the accuracy of the prediction could be very detrimental. Hence, perhaps using one the Bayesian methods may be a safer approach if evidence of a segregating QTL is yet to be confirmed.

The design, focusing primarily on selection of individuals with high and low skatole concentrations within full‐sib families, had the objective of increasing the power of identifying QTL that affect skatole in GWAS by boosting the value of long‐term linkage disequilibrium (LD) in obtaining marker–QTL associations and reducing the emphasis on LD arising from more recent family structure. Luan *et al*. (2012) showed that, in some populations, more recent family structure can be captured using linkage analysis to construct relationships and that this can account for most of the achieved accuracy from genomic evaluation. The design has immediate consequences for the results presented, as the selection introduces biases into the estimates of predicting the phenotype and the estimates of heritability, whether genomic or otherwise (Daetwyler *et al*. 2008), and consequently for the estimates of accuracy for predicting breeding values, as this prediction uses both of these parameters. The selection also has an impact on androstenone because, although this was not directly selected upon, selection was not random, as a genetic correlation (*r*_*A*_) exists between these traits (Grindflek *et al*. 2011; Strathe *et al*. 2013a,b). The latter study estimates this parameter to be 0.41 (SE = 0.14) in the Danish Landrace after accounting for selection.

For the phenotypic selection used in the design, the relative selection differential for the two traits is given by *h*_*a*_ *r*_*A*_/*h*_*s*_, where $h_{a}^{2}$ and $h_{s}^{2}$ are heritabilities for androstenone and skatole respectively. Using the parameters of Strathe *et al*. (2013a,b), the strength of the selection on androstenone is predicted to have been less, but the moderate genetic correlation is offset by the higher heritability of androstenone compared to skatole. The ranking of the methods would not be expected to be affected by the selection on skatole concentration *per se*. Had sampling been at random from the population, the expectation would be that accuracies (as reported from a cross‐validation using such data) would be reduced, given that less informative families would have been used. However, the summary of the methods as stated at the start of the discussion would be expected to remain valid, as the amount of population‐wide data increased according to the QTL/SNPs.

As outlined earlier, boar taint provides challenges for the breeder in that it is an age‐ and sex‐limited trait and destructive to measure directly. Initial attempts using selection on indirect traits, such as concentrations in the blood or size of the sex glands, were less successful than anticipated. The genetic reasons for this relative failure came from initial heritability estimates that proved to be overly optimistic and some unfavourable genetic correlations (Willeke *et al*. 1980; Sellier & Bonneau 1988; Sellier *et al*. 2000). Reducing the expression of boar taint is expected to be associated with reduced androstenone concentrations in fat and blood, but because androstenone is synthesised together with other steroids, such as androgens and estrogens (Robic *et al*. 2011), selection against taint resulted in lengthening the time to sexual maturity in pigs with low androstenone levels. However, skatole appears in fat through a relatively short metabolic pathway (Zamaratskaia & Squires 2009), which reduces the number of network interactions that may occur, and empirically, a reduction in skatole has not been associated with a negative effect on sex hormones. Therefore, skatole seems a more promising trait to use for utilising in selection.

The results from this study advance the opportunities for selection against the expression of taint as it demonstrates that genomic predictions, simultaneously utilising all SNPs for related chemical compounds, will offer opportunities to select against expression of taint that overcomes the age and sex limitations and the destructiveness of measuring the trait. Furthermore, these accuracies will increase as more data are obtained for training these genomic predictors, especially for androstenone. However, the results do not address the remaining barrier to implementing genomic evaluations in practice, which is the uncertain and possibly unfavourable genetic correlations of the expression of boar taint with other traits of value. Estimates of genetic correlations of skatole and androstenone concentrations in fat with male fertility (Strathe *et al*. 2013b) and production traits (Strathe *et al*. 2013a) have been obtained from Danish Landrace pedigree data, but the standard errors of these key parameters remain large.

Therefore, approaches for the practical application of genomics to reduce boar taint whilst managing the risk of unfavourable correlated responses are required. In all approaches, more population‐wide data will need to be collected on skatole and androstenone concentrations in fat, together with individual genotypes, to validate findings and further improve accuracy through boosting the size of the genomic training set. One approach, as mentioned above, is to prioritise selection against skatole, which may be more free from unfavourable correlations than androstenone concentrations are (Moe *et al*. 2009; Strathe *et al*. 2013a,b) and is also considered to have a greater impact on customer acceptability than androstenone (Bonneau & Squires 2004; Lee *et al*. 2005). This approach would use the Bayesian models to exploit the large QTL, which explains substantial genetic variance in the population, and obtain greater accuracy. Alongside this, androstenone concentrations could be included in routine GBLUP evaluations to accumulate more information on key genetic correlations. Furthermore, the use of GBLUP for both skatole and androstenone can be attractive for a breeding company, despite a potential loss of accuracy for skatole, estimated at 5% in this current data. This is because GBLUP likely would be used for other key traits in the breeding goal, and so additional traits evaluated with GBLUP are more easily integrated into the time‐bound, computationally demanding, multitrait evaluations that are required for effective breeding operations. In addition, the genomic predictor can be used to explore potential correlated responses by regressing detailed fertility phenotypes (e.g. age at puberty) that might be obtained only for the elite population on the genomic prediction for skatole and androstenone concentration. This is analogous to the widely used practice of regressing phenotypes on BLUP EBV as an indicator of potential correlated responses. Such approaches fulfil one of the long‐term aspirations of genomics: utilising field records from lower on the pyramid, in this case related to boar taint compounds, to provide haplotypes for direct selection at the top of the pyramid.

## Conclusion

For this dataset of a commercial Danish Landrace population, different ranges of accuracies were calculated using different methodologies of genomic selection against boar taint. For androstenone concentration, GBLUP and regression‐based methodologies performed with equal accuracy in predicting phenotypes, which was anticipated, as prior evidence suggests genetic variance is not dominated by a few QTL. In contrast, when predicting skatole concentrations, Bayesian regression methodologies had greater accuracy than did GBLUP, consistent with a large QTL known to be segregating in this population. The barriers to cost‐effective genetic selection against boar taint, arising from the age and sex limitations and destructiveness of measuring boar taint, can be removed using genomic evaluations, subject to developing a training set of adequate size. The development of predictors from field data also can assist in removing uncertainties over unfavourable genetic correlations between boar taint and other traits of value by utilising the genomic predictors in more detailed studies within elite populations. The results obtained from this study demonstrate such solutions are worthwhile considering in national breeding strategies to address the need for reliance on castration.

## Conflict of interest

The authors declare they have no conflict of interests in this research.
